# Supplementary material for: The Chromosome-Scale Genomes of Exserohilum rostratum and Bipolaris zeicola Pathogenic Fungi Causing Rice Spikelet Rot Disease
Source: J Fungi (Basel). 2023 Jan 28;9(2):177. doi: 10.3390/jof9020177 (PMC9961003; doi:10.3390/jof9020177)
Supplement: Supplementary file 1 [file jof-09-00177-s001.zip › jof-2107703-supplementary/Supplementary/Supplementary figure.pdf]

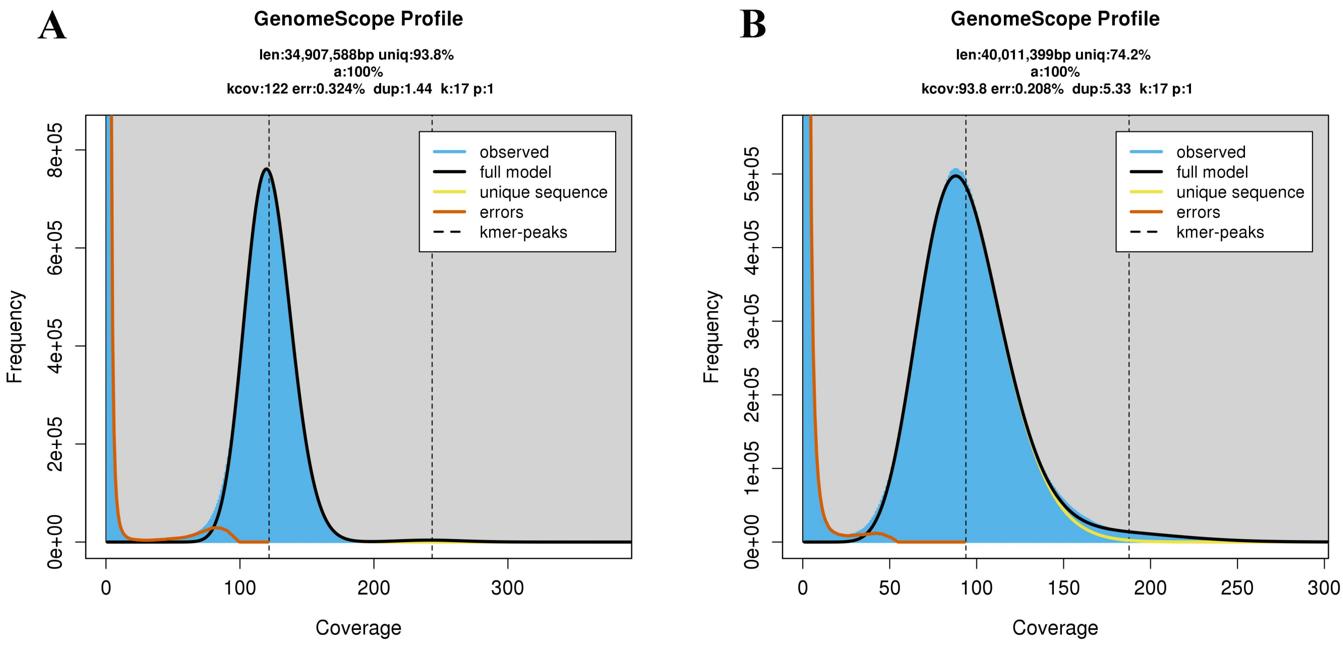

Figure S1. Assessing the genome size of *E. rostratum* LWI (**A**) and *B. zeicola* LWII (**B**).

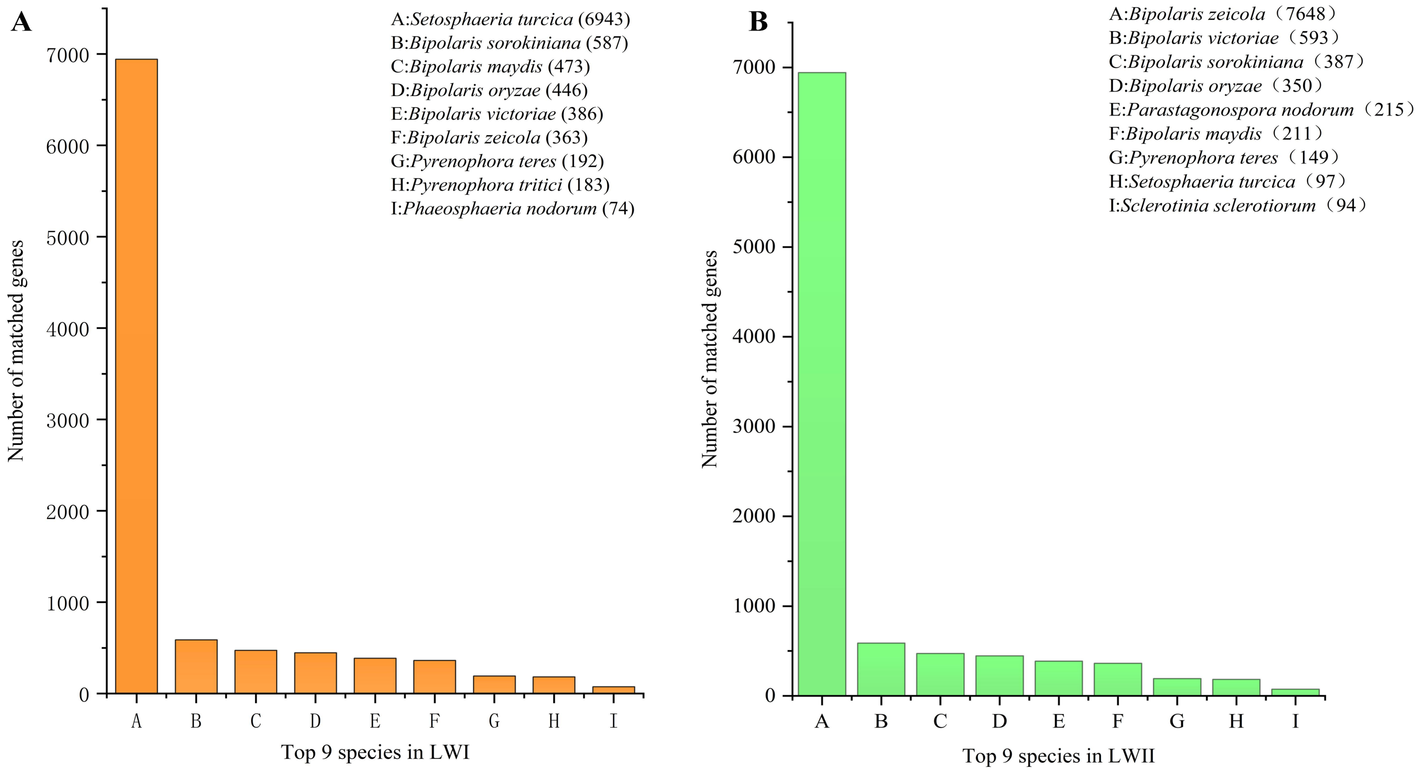

Figure S2. Predicted proteins between different fungal species using the Nr database for the *E. rostratum* LWI (**A**) and *B. zeicola* LWII (**B**) genomes.

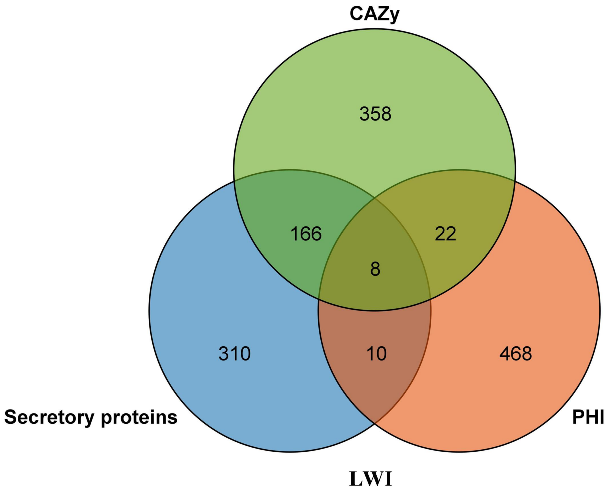

Figure S3. Intersection of annotation results of secreted protein genes, PHI genes, and CAZy genes of *E. rostratum* LWI and *B. zeicola* LWII.
